# Berberine-mediated Ferroptosis through System Xc^-^/GSH/GPX4 Axis Inhibits Metastasis of Nasopharyngeal Carcinoma

**DOI:** 10.7150/jca.90574

**Published:** 2024-01-01

**Authors:** Yao Wu, Qunying Jia, Qi Tang, Hongyu Deng, Yingchun He, Faqing Tang

**Affiliations:** 1 The First Clinical College of Traditional Chinese Medicine of Hunan University of Chinese Medicine, and Hunan Cancer Hospital, Changsha, 410007, China.; 2 Hunan Key Laboratory of Oncotarget Gene and Clinical Laboratory, The Affiliated Cancer Hospital of Xiangya School of Medicine, Central South University/ Hunan Cancer Hospital, Changsha 410013, China.

**Keywords:** BBR, GPX4, Ferroptosis, Nasopharyngeal carcinoma, Metastasis, Invasion

## Abstract

Nasopharyngeal carcinoma (NPC) is a malignant tumor that is highly prevalent in Southeast China, and its metastasis remains an unresolved clinical problem. Ferroptosis, a type of nonapoptotic cell death, is a critical pathway in tumor metastasis. Berberine (BBR), a plant alkaloid, has been explored as a potential anti-NPC metastatic agent; however, the underlying mechanisms are unknown. Here, we showed that BBR exerted its anti-metastasis role by inhibiting system Xc^-^/GSH/GPX4 axis-driven ferroptosis. The present study demonstrated for the first time that BBR induced ferroptosis in NPC cells by increasing reactive oxygen species, lipid peroxidation and cellular Fe^2+^ and that the ferroptosis inhibitors Ferrostatin-1 and Deferoxamine mesylate rescued BBR-induced NPC cell death. Moreover, the ferroptotic characteristics of BBR-treated NPC cells were observed using transmission electron microscopy. Mechanistically, system Xc^-^ (SLC7A11 and SLC3A2) and GSH levels were found to be suppressed after treatment with BBR. We demonstrated that the system Xc^-^/GSH/GPX4 axis was a critical mediator of BBR-induced ferroptosis. Furthermore, GPX4, a key inhibitor of lipid peroxidation, was greatly suppressed by BBR at both protein and mRNA levels. Molecular docking results showed a strong interaction between GPX4 and BBR. Notably, GPX4 overexpression reversed the effect of BBR-induced ferroptosis in NPC cells. Finally, BBR-mediated inhibition of NPC metastasis was validated *in vivo* using a mouse model. Taken together, our data suggest that BBR induced ferroptosis of NPC cells via suppressing the system Xc^-^/GSH/GPX4 axis, provides new insights into the mechanism of BBR anti-NPC metastasis.

## Introduction

Nasopharyngeal carcinoma (NPC) is a malignant epithelial tumor originating in the nasopharynx and is prevalent in Southeast Asia and North Africa 1. Radiotherapy alone or in combination with chemotherapy are the main treatments for NPC; however, a large number of patients die due to recurrence and the development of tumor metastasis 2. Distant metastasis contributes substantially to treatment failure and mortality in patients with NPC. Recent studies have revealed, the key role of ferroptosis in tumor metastasis. For example, lymphoid tissue protects tumor cells against ferroptosis and promotes melanoma metastasis 3, whereas induced ferroptosis suppresses tumor brain metastasis in a mouse model of breast cancer 4. These findings highlight the importance of ferroptosis in tumor growth and metastasis. Identifying new anticancer strategies and discovering new drugs that induce ferroptosis will be beneficial for improving cure rates in patients with advanced NPC.

Ferroptosis is a reactive oxygen species (ROS)- and iron-dependent programmed cell death that is distinct from apoptosis, pyroptosis, autophagy, and necrosis in morphology and molecular mechanisms, which is endogenously offset by the system Xc^-^/GSH/GPX4 axis 5. Mechanistically, system Xc^-^, a cystine/glutamate antiporter system, composed of a light-chain subunit SLC7A11 (xCT) and a heavy-chain submit SLC3A2 (CD98), is a promising target for inhibiting ferroptosis in cancer cells 6. The mediation of ferroptosis by suppressing system Xc^-^ was closely related to inhibition of cancer cell proliferation, invasion, and metastasis 7. Glutathione (GSH) biosynthesis and the selenoenzyme glutathione peroxidase (GPX4) are crucial in cell ferroptosis 8, 9. Genetic studies performed in cells and mice established GPX4 as a key regulator of ferroptosis, and conditions that culminate in GPX4 inhibition trigger ferroptosis 10. Direct or indirect GPX4 inactivation or inhibition induces a lethal lipid peroxidation process, that can be induced in certain cancer cell populations, in a controlled manner as a therapeutic approach 11. Over the past decade, ferroptosis has been implicated in the development and therapeutic response of various types of tumors 12. Although the precise mechanism of ferroptosis remains elusive, it is a new therapeutic strategy for treating cancer cells 13. Emerging evidence has shown the potential of triggering ferroptosis in cancer therapy, particularly for eradicating aggressive malignancies 14. Recent evidence suggests that NPC is intrinsically susceptible to ferroptosis 15, 16, and ferroptosis can selectively target aggressive cancer stem cells 17. This highlights a novel mechanism of this non-apoptotic cell death providing a potential strategy for NPC treatment.

Previous studies have indicated that several small-molecule agents that induce ferroptosis have great potential for the treatment of different types of cancers, including breast, pancreatic, prostate, and head and neck cancer 18. Berberine (BBR), a natural isoquinoline alkaloid from the protoberberine class, has a wide range of pharmacological activities, including antibacterial, antidiabetic, anti-inflammatory, and fungicidal properties and has shown great therapeutic efficacy in the treatment of several diseases 19-21. Previous studies have indicated that BBR can treat patients with metastatic NPC, by inhibiting metastasis 22-24. However, little is known about the molecular mechanisms underlying the anti-metastatic effects of BBR. This study demonstrates that BBR suppressed the system Xc^-^/GSH/GPX4 axis, inducing NPC cell ferroptosis and inhibiting NPC metastasis. Therefore, inducing cell ferroptosis induction may be a novel anti-metastatic mechanism of BBR in NPC.

## Materials and Methods

Animal experiments were performed in accordance with the guidelines for experimentation with laboratory animals established by The Affiliated Cancer Hospital of Xiangya School of Medicine, Central South University. All protocols were approved by the Animal Experimentation Ethics Committee of The Affiliated Cancer Hospital of Xiangya School of Medicine, Central South University (No: KNZY-202210).

### Cell culture

Human NPC cell lines S18 and 5-8F were purchased from the Institute of Biochemistry and Cell Biology of the Chinese Academy of Sciences (Shanghai, China). S18 cells were isolated from CNE-2 cells, and 5-8F cell lines were derived from CNE1 cell lines, both of which have high migration and invasion abilities. The cells were cultured in RPMI 1640 medium supplemented with 10% FBS and 100 U/ml penicillin/streptomycin at 37 °C in a humidified incubator containing 5% CO_2_. All cell lines were authenticated using short tandem repeat profiling prior to experiments.

### Reagents and antibodies

The antibodies were used for western blotting or immunohistochemistry, anti-GPX4 (ab125066, Abcam), anti-xCT (ab175186, Abcam), and anti-CD98 (ab307587, Abcam) were purchased from Abcam (Cambridge, UK). Anti-GAPDH (60004-1-Ig, Proteintech), horseradish peroxidase-conjugated goat anti-mouse IgG (SA00001-1, Proteintech), and HRP-conjugated goat anti-rabbit IgG (SA00001-2, Proteintech) antibodies were purchased from Proteintech (Wuhan, China). Necrostatin-1 (Nec-1) (4311-88-0, Selleck), Z-VAD-FMK (Z-VAD) (187389-52-2, Selleck), Chloroquine (CQ) (54-05-7, Selleck), Deferoxamine mesylate (DFO) (138-14-7, Selleck), and Ferrostatin-1 (Fer-1) (347174-05-4, Selleck) were purchased from Selleck (Shanghai, China). BBR (10006427; purity ≥ 95%) was purchased from Cayman Chemicals (Ann Arbor, Michigan, USA).

### Cell counting kit-8 (CCK-8) assay

Cell proliferation was measured using the CCK-8 assay, according to the manufacturer's instructions (Bs350A; Biosharp, Anhui, China). Briefly, the S18 and 5-8F cells were treated with different BBR concentrations for 12, 24, and 48h. CCK8 test was used to measure cell viability.

### Detection of Lactate Dehydrogenase (LDH)

The LDH assay was performed as previously reported 25. Briefly, S18 and 5-8F cells were seeded in 6-well plates and treated with BBR at 0-160 μM concentrations at 37°C for 24 h. Media for the LDH assay was collected using the LDH Cytotoxicity assay kit (C0016, Shanghai Biyuntian Biological Co., Shanghai, China).

### Transwell and wound-healing assays

Transwell and wound healing assays were performed as previously described 26. Cell migration and invasion assays were performed using chambers according to the manufacturer's instructions (Corning Costar; 3422; Shanghai, China). Briefly, S18, and 5-8F cells were placed in the uncoated (migration assay) or Matrigel-coated (BD Biosciences, New Jersey, USA) upper chamber (invasion assay) treated with BBR at 40 or 80 μM. After incubation, the cells that migrated to the bottom surface were stained with crystal violet. For the scratch wound migration assay, a scratch wound was made using a pipette tip, and the wounds were photographed under a phase-contrast microscope (Olympus, Japan).

### Colony formation assays

Colony formation assays were performed as previously reported 27. Briefly, S18 and 5-8F cells were plated in 6-well plates for 1 week. After fixation and staining, the colonies were imaged and counted.

### Flow cytometry of cell death distribution

Flow cytometry of Cell assay was performed as described previously 28. Briefly, cells were seeded in 6-well plates and treated with BBR at 80 μM concentrations at 37 °C for 12, 24, and 48h. The cells were collected and washed with phosphate buffered saline (PBS). Finally, 10 μl Annexin V-FITC and 5 μl PI were added into the cells at 37 ℃ temperature for 15 min. The quadrant distribution of cell death was measured by flow cytometry. Annexin V-FITC Apoptosis Detection Kit (C1062M; Shanghai Biyuntian Biological Co.).

### Transmission Electron Microscopy (TEM)

TEM was performed as previously reported 29. Briefly, the cells were seeded into 100 mm cell culture dishes and exposed to BBR for 12 h. Cells were collected, washed with PBS, and fixed with 2.5% glutaraldehyde. The samples were then pretreated according to standard procedures, and images were acquired using a transmission electron microscope (Hitachi, Tokyo, Japan).

### Measurement of ROS

The ROS assay was performed as described previously 30. Briefly, the peroxide-sensitive fluorescent probe DCFH-DA (S0033S; Shanghai Biyuntian Biological Co.) was used to detect intracellular ROS according to the manufacturer's instructions.

### Malondialdehyde (MDA) assay

The MDA assay was performed as described previously 31. Briefly, MDA was detected and normalized based on protein concentration according to the manufacturer's instructions (S0131S, Shanghai Biyuntian Biological Co.).

### GSH assay

GSH assay were performed as described previously 32. Briefly, total quantities of GSH were measured using a GSH and GSSG assay kit (S0053, Shanghai Biyuntian Biological Co.) in accordance with the manufacturer's instructions. GSH content was evaluated by comparison with a standard curve of GSH.

### Detection of Fe^2+^ ions assay

Fe^2+^ ions were detected as previously reported 33. Briefly, the contents of Fe^2+^ in S18 and 5-8F cells were determined according to the manufacturer's instructions (MX4559-24UG; Shanghai Maokang Biotechnology Co.). FerroOrange (1 μM, an intracellular Fe^2+^ ions probe) dispersed in serum-free medium was added to the cells, and cells were incubated for 30 min in a 37 °C incubator, and cells were then collected. Finally, cell fluorescence was observed using a fluorescence microscope.

### Measurement of lipid reactive oxidative species

The lipid ROS assay was performed as previously described 34. Briefly, cells were treated as indicated and were then incubated for 1 h with 50 μM of lipid peroxidation sensor BODIPY 581/ 591 C11 (Invitrogen) and was analyzed using a flow cytometer.

### Quantitative real-time PCR (qRT-PCR)

qRT-PCR was performed as previously reported 35. Briefly, total RNA was extracted from the cells using the Total RNA Kit II reagent (Omega). cDNA was prepared using reverse transcriptase. mRNA expression was determined using the SYBR Green Master Mix (4309155; Thermo Fisher Scientific) on a LightCycler® 480 Instrument II (Roche Life Science). GAPDH was used for normalization. The primers used in this study are listed in Table 1. The transcript levels were analyzed using the 2^-△△Ct^ method.

### Western blotting (WB) analysis

WB assay was performed as previously reported 36. Briefly, the protein samples were denatured, subjected to sodium dodecyl sulfate-polyacrylamide gel electrophoresis (SDS-PAGE), and transferred to PVDF membranes (Millipore). The membranes were immunoblotted with specific primary antibodies and horseradish peroxidase-conjugated secondary antibodies and visualized using SuperSignal West Dura Extended Duration Substrate (Thermo Pierce).

### Lentivirus transfection

Stably transfected cells were generated as described previously 37. Briefly, lentiviral vectors including GPX4 (LV-GPX4) and the Negative Control (LVCON335), were purchased from Genechem Co. (Shanghai, China). The HEK293T packaging cells were transfected with 10 μg lentiviral vectors using the calcium phosphate method for lentivirus production. After transfection, lentiviruses were collected from the culture supernatants. NPC cells were infected with lentiviruses and selected using puromycin (5 μg/mL). The treated cells were harvested after selection and subjected to western blot analysis.

### Animal studies

Animal experiments were performed as described previously 38. Briefly, all *in vivo* experiments were performed on female BALB/c nude mice aged 4-6 weeks. The mice were obtained from the Hunan SJA Laboratory Animal Co. (Changsha, China) and housed in a room at a controlled temperature. For the metastasis model, the nude mice were randomly divided into groups (n=5 for each group); A total of 2 × 10^6^ S18 cells were suspended in 100 μl PBS and then injected into the tail veins of mice. On day 5 after injection, the mice were randomly divided into two groups (n=5 per group): control and BBR (15 mg/kg/day). After 30 days, metastasis was measured and quantified by *ex vivo* bioluminescent imaging using AniView100 Multi-mode *In vivo* Animal Imaging The excised tissues were fixed in 10% neutral buffered formalin or snap-frozen.

### Immunohistochemistry

Immunohistochemical analyses were performed as previously reported 39. Briefly, tumor tissues were fixed, paraffin-embedded, and sectioned (4 μM). Deparaffinized and rehydrated sections were subjected to antigen retrieval using sodium citrate buffer. After incubation with 10% normal goat serum for 1 h, the sections were incubated with primary antibody overnight at 4 °C. Subsequent procedures were performed according to the manufacturer's instructions (Pinofi Biotechnology Co., Ltd Wuhan, China).

### Molecular docking

The initial three-dimensional geometric coordinates of the X-ray crystal structure of GPX4 (PDB code: 2OBI) were downloaded from the Protein Databank (https://www.rcsb.org/), and molecular docking was employed to estimate the interaction between BBR and GPX4 pockets during screening using the CDOCKER protocol 40.

### Statistical analysis

Data are shown as the mean and standard error of the mean and were analyzed using GraphPad Prism software (version 8.0). One-way analysis of variance was used for statistical comparisons among groups. Statistical significance was set at *P* < 0.05.

## Results

### BBR suppresses cell proliferation and induces cell death in S18 and 5-8F NPC cells

**Figure 1:**
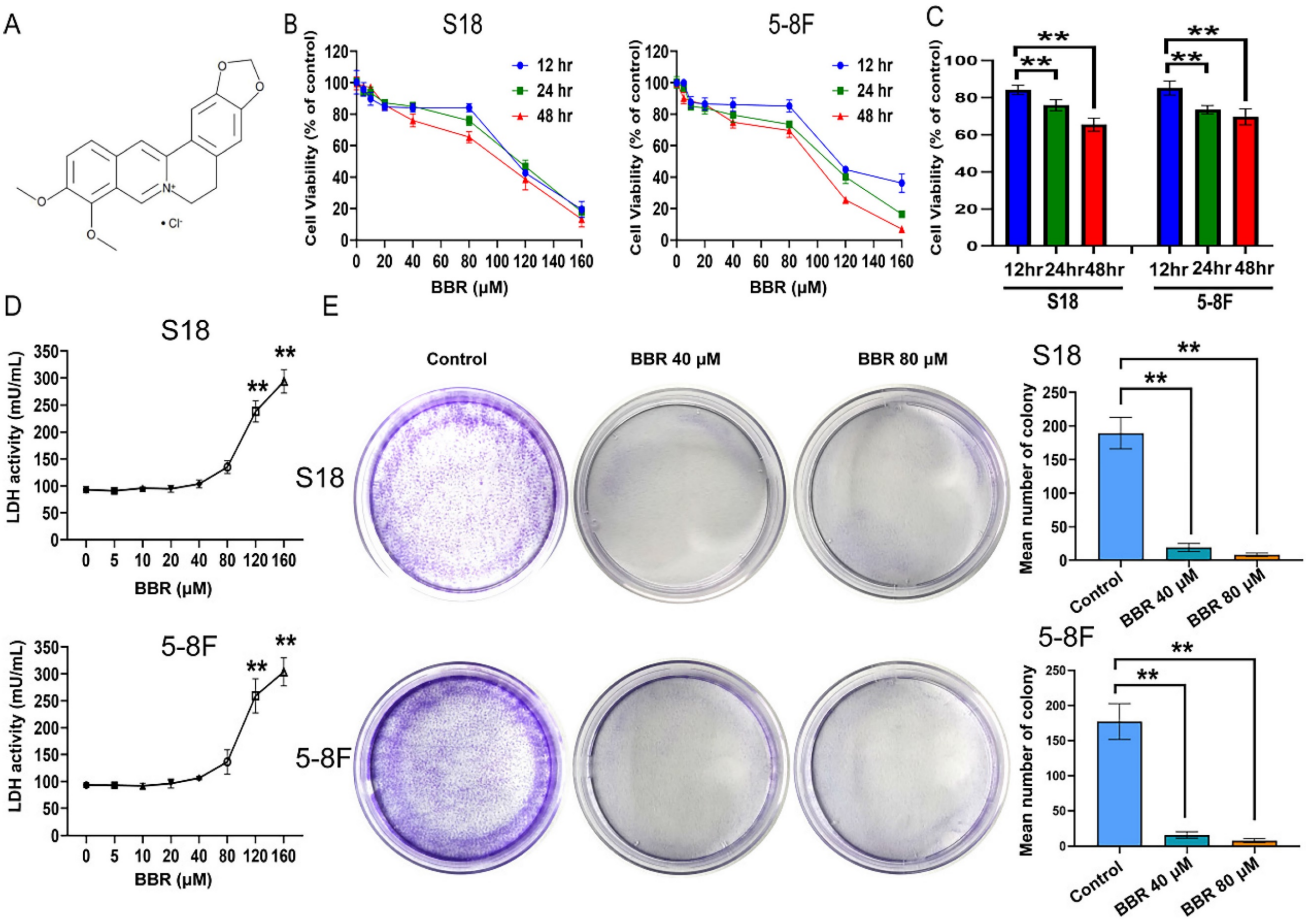
** BBR mediates cell growth suppression in S18 and 5-8F cells.** (A) Berberine chemical structure. (B) S18 and 5-8F cells were treated with BBR at 5, 10, 20, 40, 80, 120, 160 μM for 12, 24 or 48 h, and subjected to CCK-8 assay for cell viability. (C) S18 and 5-8F cells were treated with BBR at 80 μM for 12, 24 or 48 h, and subjected to CCK-8 assay for cell viability. (**P < 0.01 versus 12hr). (D) After BBR treatment for 24 h, LDH was detected with LDH assay. (**P < 0.01 versus BBR 80 μM). (E) cell growth was tested with cell colony growth. Quantitative analyses of colony numbers are shown. (**P < 0.01 versus control group).

BBR is an isoquinoline derivative alkaloid, and its chemical structure is shown in Fig. 1A. In this study, the inhibitory effect of BBR on NPC cells was determined by treating S18 and 5-8F cells with various concentrations of BBR for 12, 24, and 48 h, followed by a CCK-8 assay. The results showed that BBR inhibited the growth of 5-8F and S18 cells in a dose- and time-dependent manner (Fig. 1B and C). The LDH in the cell culture media was detected, and the data showed that LDH values were low at 0-80 µM while significantly increased at a concentration of > 80 μM BBR (Fig. 1D). These results indicated that < 80 μM was non-cytotoxic concentrations of BBR for NPC cells. Therefore, < 80 μM was applied in all subsequent experiments. To further determine the antiproliferation effect of BBR, a colony formation assay was performed. The results showed that BBR treatment significantly suppressed 5-8F and S18 cell colony formation at 40 and 80 µM (Fig. 1E), indicating that BBR inhibited NPC cell proliferation. Wound-healing assays were performed to investigate the effect of BBR on cell migration.

**Figure 2:**
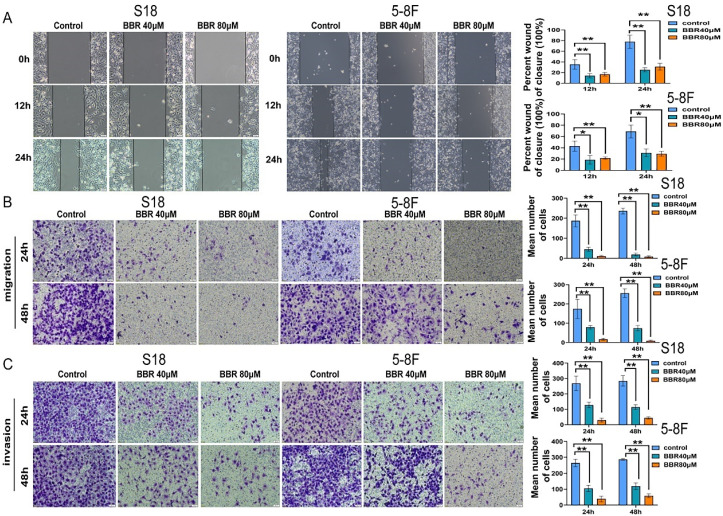
** BBR mediates cell migration and invasion suppression in S18 and 5-8F cells.** (A) Wound-healing assay was used to detect cell migration. Representative pictures of S18 and 5-8F cells cultured with the different concentrations of BBR in 24 h or 48 h, the relative migration values were presented as the means ± S.E.M. (B-C) Transwell migration and invasion assays were used to detect the migratory and invasive capabilities of S18 and 5-8F cells treatment with 40 and 80 μM, the number of migrated and invaded cells were presented as the means ± S.E.M. All experiments were done more than three times independently and statistically analyzed with one-way analysis of variance (*P < 0.05, **P < 0.01 versus control group).

**Figure 3:**
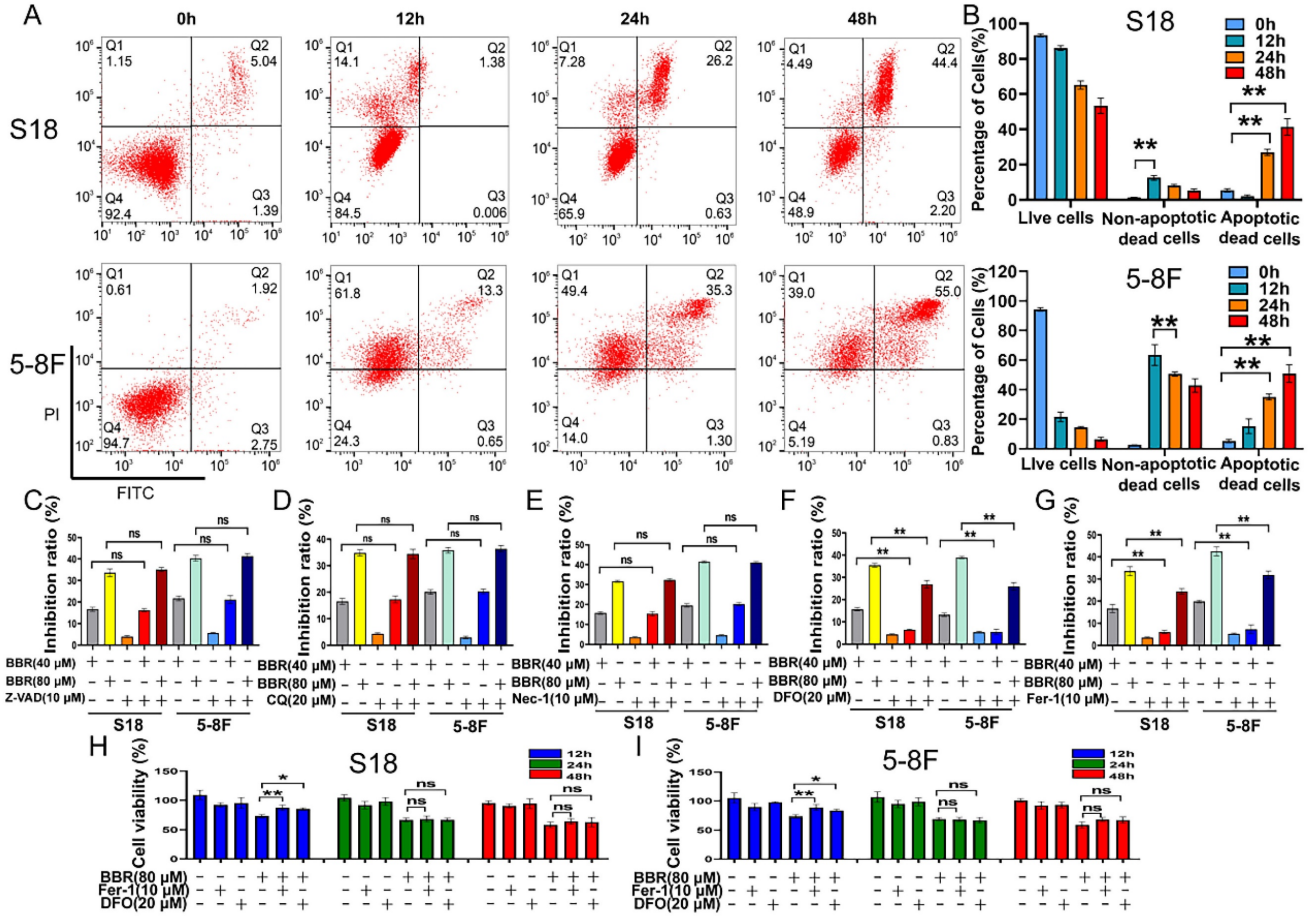
** BBR induces cell death in S18 and 5-8F cells.** (A-B) Representative results of annexin V/FITC/PI staining and quantitative analysis, (** P < 0.01 versus control group). (C-G) S18 and 5-8F cells were treated with BBR with or without Z-VAD-FMK, CQ, Nce-1, DFO, Fer-1 for 12 h, and the inhibition of growth was assayed, (** P < 0.01, ns means no significance versus control group). (H-I) Cell viability in S18 and 5-8F cells treated with BBR, DFO and Fer-1 was detected by CCK-8 assay, (*P < 0.05, **P < 0.01, ns means no significance versus control group).

The migration of 5-8F and S18 cells in the BBR group was significantly lower than that in the control group (Fig. 2A). Next, a Transwell assay was performed to further confirm the inhibitory effect of BBR on NPC cell migration. The migration and invasion of cells in the BBR group were significantly reduced after exposure to BBR for 24 and 48 h (Fig. 2B and C). Next, to investigate BBR-mediated cell death, Annexin V-FITC/PI staining was performed on the BBR-treated cells by flow cytometry. As expected, a high percentage of dead cells was observed in S18 and 5-8F NPC cells after with BBR treatment; however, the overall apoptosis was lower at 12 h (Fig. 3A and B). These data imply that apoptosis may not be the predominant pattern of cell death induced by BBR at 24 h and that various cell death mechanisms may exist in BBR-induced NPC death.

### Ferroptosis contributes to BBR-induced cell death in S18 and 5-8F NPC cells

To evaluate BBR-induced cell death, we used specific inhibitors, including Z-VAD (pan-caspase inhibitor), chloroquine (CQ, an autophagy inhibitor), necrostatin-1 (Nec-1, a necroptosis inhibitor), and the ferroptosis inhibitors deferoxamine (DFO) and ferrostatin (Fer-1). The results showed that Nec-1, Z-VAD, and CQ could not rescue BBR-induced cell death caused by BBR on S18 and 5-8F cells (Fig. 3C-E); however, DFO and Fer-1 significantly rescued BBR-induced cell death at 12 h (Fig. 3F and G). These results suggested that BBR induces NPC cell death via ferroptosis. To further confirm BBR-mediated ferroptosis, BBR-treated cells were treated with DFO and Fer-1 for 12, 24, and 48 h. DFO and Fer-1 could significantly rescue cell viability in the BBR-treated cells at 12 h, but not at 24 and 48 h (Fig. 3H and I). These results indicate that ferroptosis might be the predominant cell death program for BBR-induced NPC cell death after 12 h.

**Figure 4:**
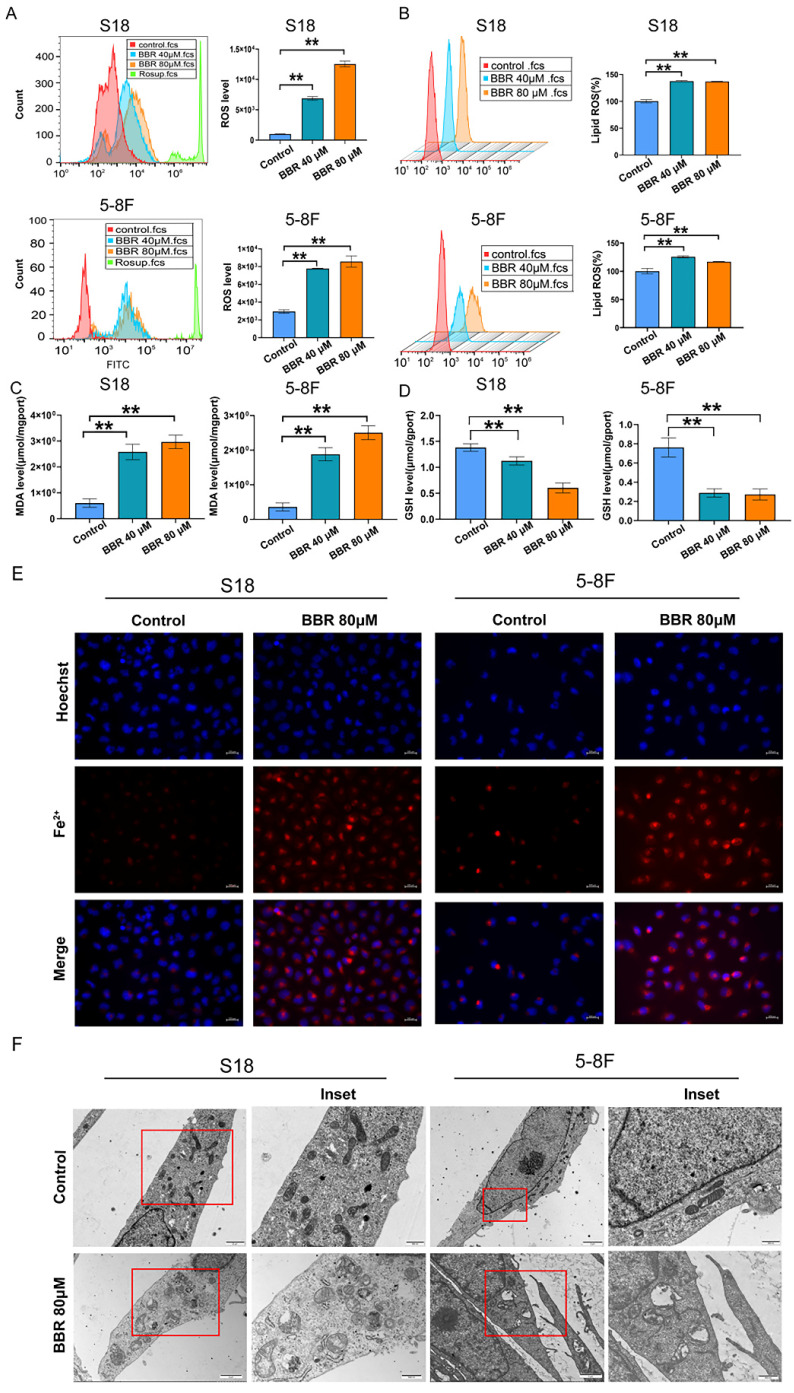
** BBR induces ferroptosis in S18 and 5-8F cells.** (A) The cellular ROS level in S18 and 5-8F cells treated with BBR was analyzed by a flow cytometer, Rosup was ROS positive control, (*P < 0.05, **P < 0.01, versus control group). (B) The cellular lipid peroxidation in S18 and 5-8F cells treated with BBR level was detected by staining with C11-BODIPY and quantitative analysis (** P < 0.01 versus control group). (C) Intracellular MDA levels in S18 and 5-8F cells treated with BBR (** *P* < 0.01 versus control group). (D) Intracellular GSH levels in S18 and 5-8F cells treated with BBR (** *P* < 0.01 versus control group). (E) Intracellular Fe^2+^ iron in S18 and 5-8F cells treated with BBR was determined using the FerroOrange. (F) Transmission electron microscopy (TEM) was used to observe ferroptosis in S18 and 5-8F cells, scale bar represents 2 µM and 500 nm.

The reports documented ROS accumulation, GSH depletion, lipid peroxidation, and redox-active iron overload are critical events in the ferroptosis process 41. The levels of intracellular ROS, GSH, and the oxidative stress marker MDA were also measured. As expected, increased intracellular total ROS and lipid peroxidation levels (Fig. 4A and B) and decreased GSH levels (Fig. 4D) were observed following BBR treatment. BBR treatment upregulated the MDA levels in S18 and 5-8F NPC cells (Fig. 4C). Additionally, FerroOrange was used to monitor the intracellular labile Fe^2+^ levels, which were quenched upon binding to iron. The results showed that BBR treatment dramatically decreased Fe^2+^ levels in S18 and 5-8F NPC cells (Fig. 4E), indicating ferroptosis. We examined the effects of BBR on the morphological characteristics of ferroptotic cells. A unique morphological characteristic of ferroptosis is a decrease in mitochondrial volume and an increase in membrane density 42. The ferroptosis mitochondrial characteristics, swelling, decreased cristae, mitochondrial vacuolization, and increased membrane density were observed in BBR-treated cells under TEM (Fig. 4F). These results indicated that ferroptosis plays a vital role in BBR-induced cell death in S18 and 5-8F NPC cells.

### Blocking ferroptosis reverses BBR-mediated NPC cell death

**Figure 5:**
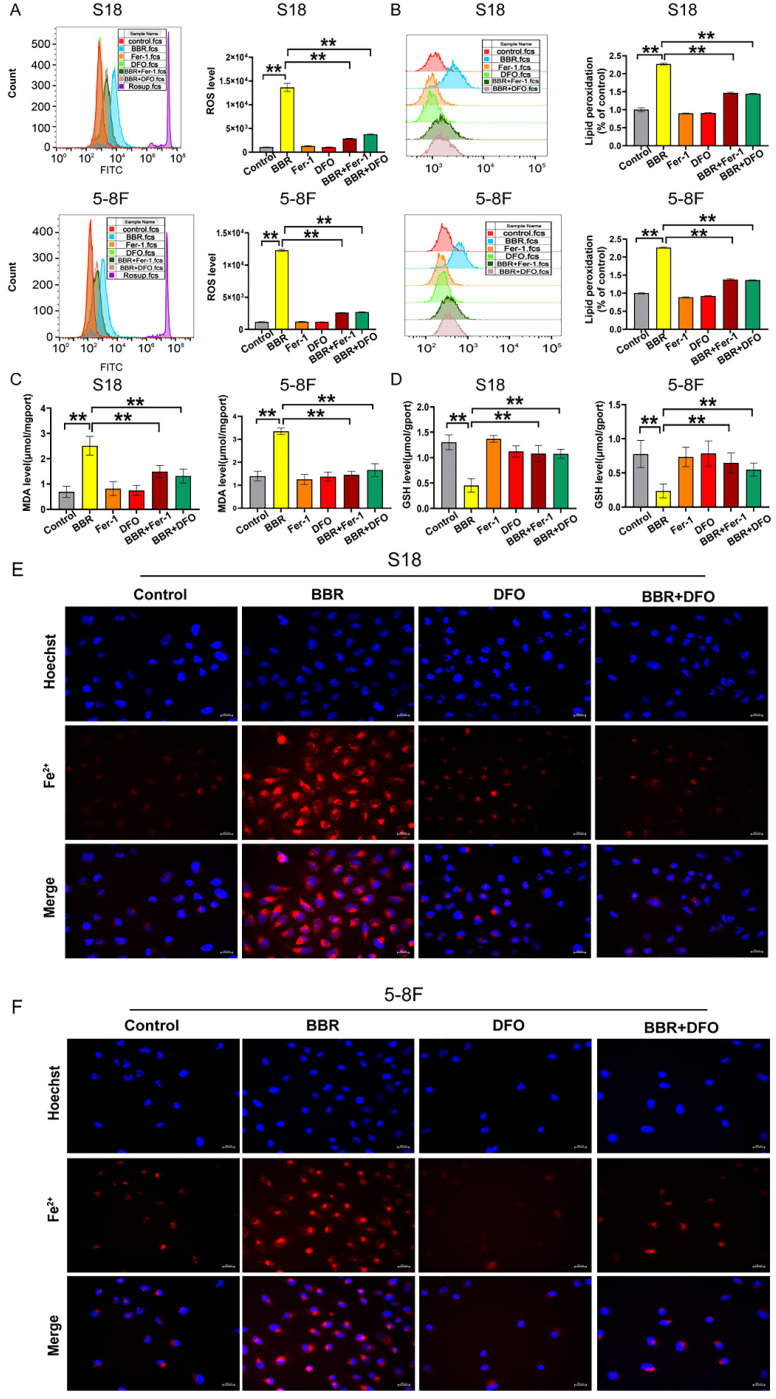
** DFO and Fer-1 blocks BBR-mediated NPC cell ferroptosis.** (A) The cellular ROS level in S18 and 5-8F cells treated with BBR, DFO and Fer-1 was analyzed by a flow cytometer, Rosup was ROS positive control, (*P < 0.05, **P < 0.01, versus control group). (B) The cellular lipid peroxidation level in S18 and 5-8F cells treated with BBR, DFO and Fer-1 was detected by staining with C11-BODIPY and quantitative analysis (** P < 0.01 versus control group). (C) Intracellular MDA levels in S18 and 5-8F cells treated with BBR (** P < 0.01 versus control group). (D) GSH levels in S18 and 5-8F cells treated with BBR (** *P* < 0.01 versus control group). (E-F) Fe^2+^ iron in S18 and 5-8F cells treated with BBR and DFO was determined using the FerroOrange.

To further determine the role of ferroptosis in BBR-induced NPC cell death, the ferroptosis inhibitors, DFO and Fer-1 were used to block ferroptosis and ROS, MDA, and GSH were detected in BBR-treated cells. As expected, DFO and Fer-1 significantly decreased the BBR-induced ROS, lipid peroxidation (Fig. 5A and B), and MDA (Fig. 5C) levels, while significantly increasing the BBR-reduced GSH levels (Fig. 5D). DFO rescued the BBR-induced acumulation of iron (Fig. 5E and F). These results indicate that BBR induces NPC cell ferroptosis, which may be the primary mechanism underlying the anti-NPC effect of BBR.

### System Xc^-^/GSH axis in BBR-induced NPC cell ferroptosis

**Figure 6:**
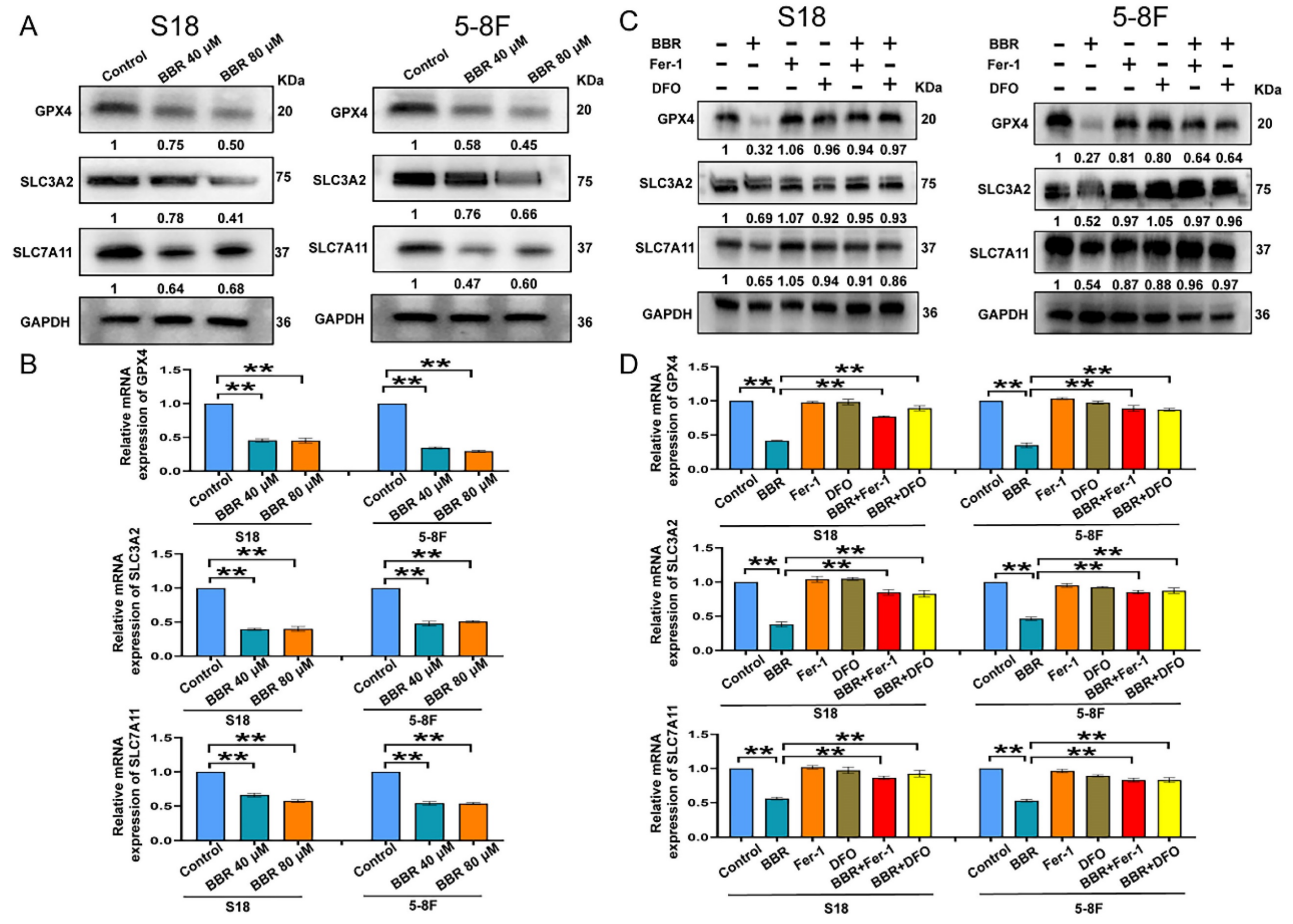
** System Xc^-^/GSH axis is involved in BBR-induced NPC cell ferroptosis.** (A) The expressions of GPX4, SLC3A2, SLC7A11 protein in S18 and 5-8F cells treated with BBR was examined by western blotting. (B) The expression of GPX4, SLC3A2 and SLC7A11 mRNA in S18 and 5-8F cells treated with BBR was examined by qRT-PCR. (C) The expression of GPX4, SLC3A2 and SLC7A11 protein in S18 and 5-8F cells treated with BBR, DFO and Fer-1 was examined by western blotting. (D) The expression of GPX4, SLC3A2 and SLC7A11 mRNA in S18 and 5-8F cells treated with BBR, DFO and Fer-1 was examined by qRT-PCR.

Ferroptosis is triggered by lipid peroxidation and is tightly regulated by SLC7A11 and SLC3A2, the key components of the cystine-glutamate antiporter 43. Downregulation of GPX4 can directly or indirectly trigger ferroptosis as a result of the inhibition of lipid peroxidation inhibition 44. To identify the specific molecular mechanism of BBR-induced ferroptosis, NPC cells were treated with various concentrations of BBR and the mRNA and proteins levels of GPX4, SLC7A11, and SLC3A2 were detected. The protein levels of GPX4, SLC7A11, and SLC3A2 decreased following BBR treatment in a dose-dependent manner in S18 and 5-8F NPC cells (Fig. 6A). Meanwhile, the mRNA levels of GPX4, SLC7A11, and SLC3A2, also decreased (Fig. 6B). These results revealed that GPX4 might be a main molecule and system Xc^-^/GPX4 axis plays an important role in BBR-induced NPC cell ferroptosis. Moreover, DFO and Fer-1 rescued BBR-induced expression of ferroptosis proteins and mRNA (Fig. 6C and D).

### Overexpression of GPX4 reverses the effect of BBR-induced cell ferroptosis

**Figure 7:**
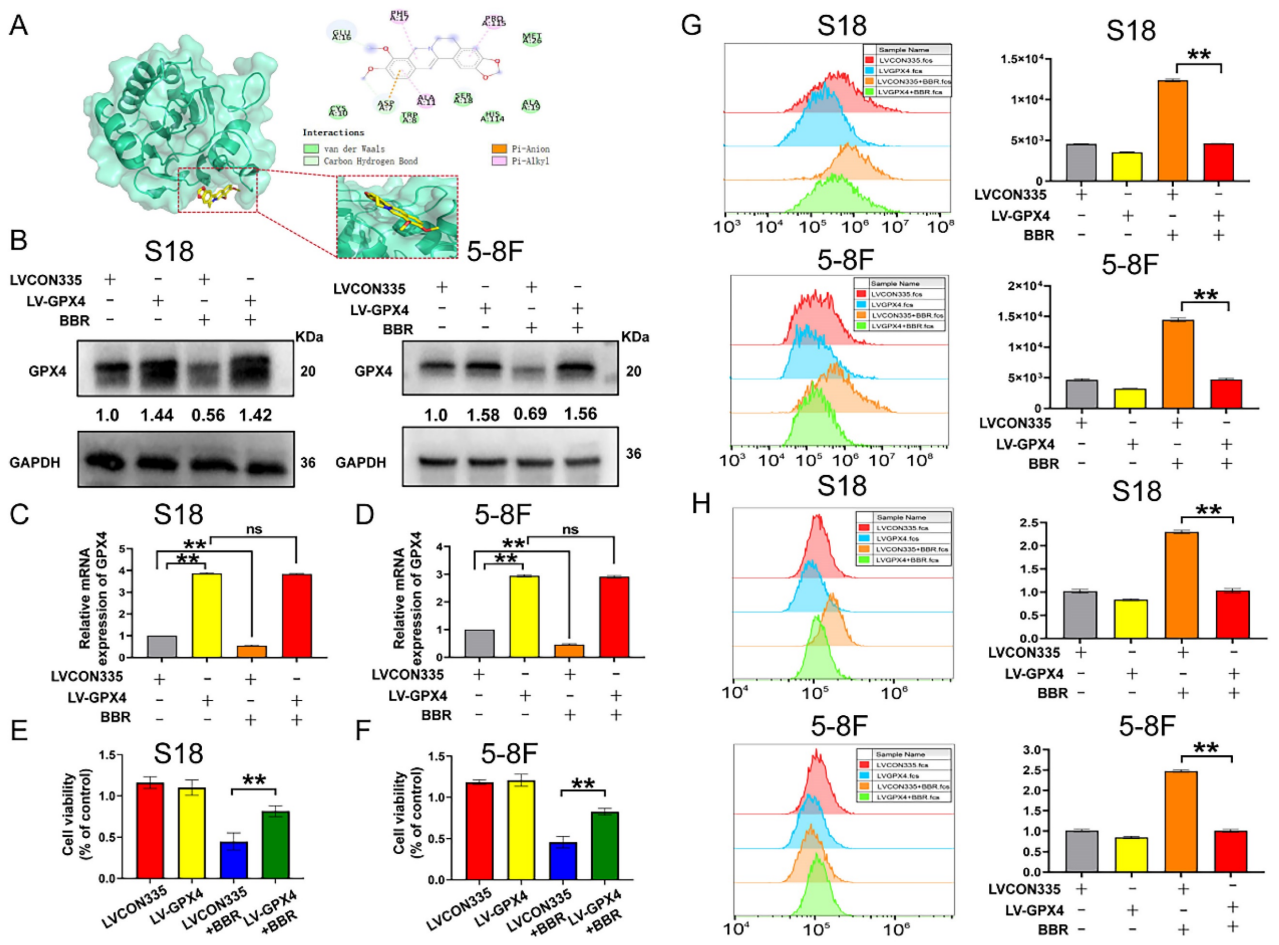
** Overexpresion of GPX4 reverses the effect of BBR-induced cell ferroptosis.** (A) CDOCKER predicted binding mode of BBR with GPX4. (B) GPX4 protein expressions were detected in S18 and 5-8F cells after overexpression of GPX4. (C-D) The GPX4 mRNA expression were detected in S18 and 5-8F cells after overexpression of GPX4 (** P < 0.01, ns means no significance versus control group). (E-F) Cell viability in S18 and 5-8F cells after overexpression of GPX4 and treated with BBR, DFO and Fer-1 was detected by CCK-8 assay (** P < 0.01, ns means no significance versus control group). (G) The cellular ROS level in S18 and 5-8F cells after overexpression of GPX4 and treated with BBR was analyzed by a flow cytometer, (**P < 0.01, versus control group). (H) The cellular lipid peroxidation level in S18 and 5-8F cells after overexpression of GPX4 and treated with BBR was analyzed by a flow cytometer, (**P < 0.01, versus control group).

Since GPX4 plays a crucial role in the process of NPC cell ferroptosis, we further predicted the potential targets for BBR induced ferroptosis using AutoDock Vina and found that BBR may target GPX4. BBR was found to form a pi-alkyl bood with PHE-17, PRO-115, and ALA-11, a pi-anion with ASP-7, and carbon hydrogen bonds with ASPA-7, GLUA-16, and ASP-34 (Fig. 7A). We validated the role of GPX4 in BBR-induced ferroptpsis of NPC cells. GPX4 was overexpressed in S18 and 5-8F NPC cells by lentivirus transfection, and ferroptosis was observed (Fig. 7B and C). Lipid peroxidation and ROS assays showed that the BBR-induced effect on NPC cell ferroptosis was substantially decreased following GPX4 overexpression (Fig. 7G and H). The inhibitory effect of BBR on NPC cell viability was partially reversed by over-expressed GPX4 (Fig. 7E and F). These results further confirmed that GPX4 contributes to BBR-induced ferroptosis in NPC cells, suggesting that GPX4 plays a crucial role in the anti-NPC effects of BBR.

### BBR inhibits NPC metastasis *in vivo*

**Figure 8:**
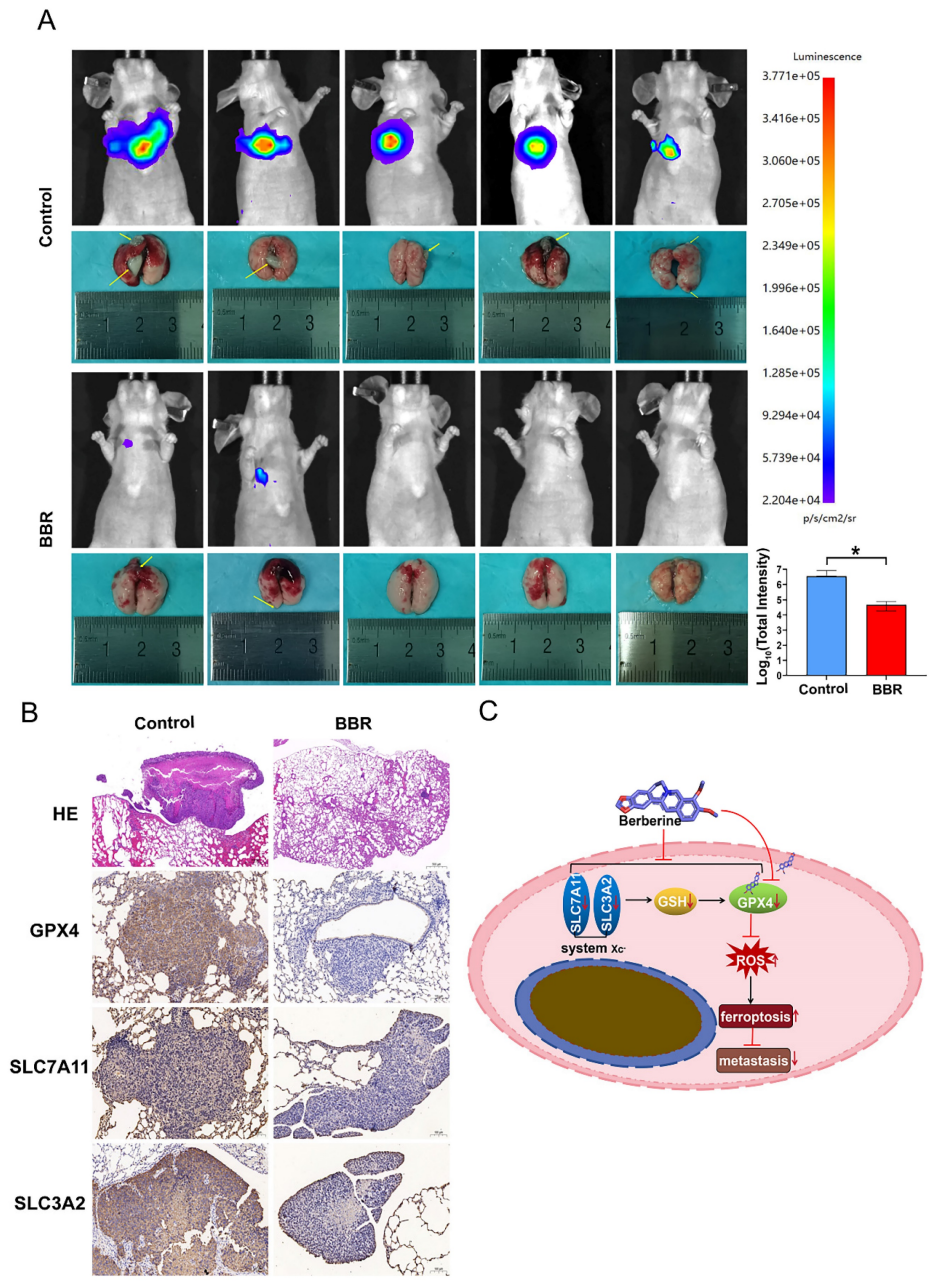
** BBR inhibits NPC metastasis in mice.** (A) A total of ten nude mice were randomly allocated to the control group (n = 5) or the BBR group (n = 5). The S18 cells were injected into the tail vein of nude mice. The mice were subjected to BBR (15mg/kg/day) by intraperitoneal injection for 30 day (n = 5). And the luminescence intensity of lung metastases was analyzed *in vivo* using an *in vivo* small animal imaging system, (*P < 0.05, versus control group). (B) Hematoxylin-eosin (HE) staining was used to detect the percent of tumor metastases per lung. The expression of GPX4, SLC3A2 and SLC7A11 in NPC lung metastatic tissues and NPC primary tissues were measured by IHC staining. (C) The diagram showing BBR induced NPC cell ferroptosis through the system Xc^-^/GSH/GPX4 axis.

To evaluate the anti-metastatic effect of BBR on NPC cells *in vivo*, 2×10^6^ S18 cells with vector luciferase were injected into the tail veins of nude mice and then treated with BBR. Thirty days after injection, the fluorescence intensity was detected in the mice, and their lungs were excised for examination. The results showed that BBR-treated group had fewer metastatic lesions than the control group (Fig. 8A). Immunohistochemical staining of pulmonary metastases showed that GPX4, SLC7A11 and SLC3A2 proteins were elevated compared with the control, which was consistent with the *in vitro* results, of the downregulated expression of GPX4, SLC7A11, and SLC3A2 in the BBR-treated group (Fig. 8B). Taken together, these findings revealed that BBR markedly inhibited NPC metastasis both *in vitro* and *in vivo*.

## Discuss

NPC is a metastasis-prone malignancy with the highest metastatic rate among head and neck cancers 45. More than 70% of patients present with locoregionally advanced disease, and distant metastasis is the main cause of treatment failure 46, 47. Although radiotherapy, combined with chemotherapy are effective therapies for NPC, they have side effects, including hearing loss and cranial nerve damage, resulting in NPC patients being unable to complete these therapies, leading to therapeutic failure 48. Therefore, it is critical to identify drugs that targeting tumor with low toxicity and high efficiency that target tumors to treat NPC.

Chinese herbal medicines have a long history of inhibiting tumor cell proliferation, invasion, and metastasis 49. Coptidis Rhizoma is a well-konwn traditional Chinese medicine used to treat various diseases including NPC 50. Coptidis Rhizoma has been widely used in Chinese medicine to treat various inflammatory diseases and cancer for thousands of years 51. BBR, a natural product derived from Coptidis Rhizoma, has multiple pharmacological functions. Reports have shown that the anticancer effects of BBR are mediated through the regulation of different molecular pathways that induce apoptosis, autophagy, and cell cycle arrest 52. Previous studies have suggested that BBR effectively induced NPC cell death and inhibited metastasis 22. Although BBR has been widely used clinically as an antitumor treatment, the potential mechanisms of its anticancer effects on NPC cells remain unclear.

Ferroptosis plays an important role in cancer therapeutic strategies 53. GPX4 inactivation and ROS accumulation are vital regulators of ferroptosis 54. Recent studies have shown that ferroptosis induces cell death and suppresses cancer metastasis 55, 56, whereas inhibiting ferroptosis by increasing GPX4 and reducing intracellular ROS levels can facilitate cancer metastasis 57, 58. Thus, inhibition of ferroptosis is considered one of the primary reasons for the uncontrolled proliferation and metastasis of tumor cells 59. Hence, driving ferroptosis is widely believed to be an effective method for treating tumor metastasis 60.

Currently, ferroptosis-inducing drugs are attracting increasing attention for cancer treatment and will hopefully provide a potential strategy for cancer therapy. Yi et al. found that BBR significantly decreased gastric carcinoma cell viability and induced cell death. In contrast ferroptosis inhibitors diminished the promoting effect of BBR on cell death, and BBR promoted autophagy to activate ROS-mediated ferroptosis in gastric carcinoma cells 61. Previous studies have not demonstrated the anticancer effects of BBR on ferropotosis in NPC cells. Therefore, it is important to supplement the research in this area and further investigate the molecular mechanism underlying the anti-metastatic effects of BBR. In this study, we observed the effects of BBR on NPC cells both *in vitro* and *in vivo*. The results showed that BBR could inhibit cell proliferation, block up the system Xc^-^/GSH/GPX4 axis induce ferroptosis, and suppress migration in NPC cells.

In the present study, we report for the first time that BBR exerts its anticancer activity by inducing ferroptosis and inhibiting the proliferation and metastasis of NPC cells. The effects underlying the antiproliferative and metastatic abilities of BBR in NPC cells were verified. Tumor metastasis of NPC cells was reduced in a mouse lung xenograft model after BBR administration. Previous studies have indicated that BBR, through the regulation of apoptosis and autophagy inhibits metastasis and invasion 62.

However, we found that treatment with Z-VAD-FMK, CQ, or necrostatin-1 did not protect against BBR-induced cell death in NPC cells, indicating that other forms of cell death may have occurred. Ferroptosis was detected in NPC cells treated with BBR. These data suggested that BBR significantly triggered the molecular characteristics of ferroptosis in S18 and 5-8F cells, including the depletion of GSH, lipid peroxidation, and accumulation of ROS, MDA, and iron. We observed changes in the characteristics of ferroptosis in the mitochondria of NPC cells treated with BBR, including swollen mitochondria with fractured cristae and increased membrane density. In addition, treatment with the ferroptosis inhibitors, Fer-1 and DFO reduced BBR-induced ferroptosis in S18 and 5-8F cells. DFO and Fer-1 significantly rescued BBR-induced cell death after only 12 h, implying that BBR may elicit different types of cell death at different time points. These data indicated that BBR promotes ferroptosis, which is beneficial for NPC treatment.

During ferroptosis, cysteine glutamate antiporters SLC7A11 and SLC3A2 are inhibited, resulting in decreased intracellular cystine intake, affecting intracellular and suppressing the activation of GPX4 63. GPX4 is one of the most important antioxidant enzymes attenuating ferroptosis 64. In this study, BBR inhibited the expression of GPX4, SLC7A11, and SLC3A2, which in turn induced ROS accumulation and lipid peroxidation in NPC cells, aggravating ferroptosis, as confirmed by Fer-1 and DFO assays. Molecular docking was used to explore the interaction of GPX4 with BBR at molecular structure, showing a strong combination of molecular bonds, implying that BBR directly acts on GPX4 to decrease its function. Overexpressed of GPX4 reversed BBR-induced NPC cell ferroptosis, and the inhibitory effect of BBR was partially reversed by overexpressing of GPX4. Our findings provide evidence that BBR might function as a GPX4 inhibitor in treating NPC patients, and the system Xc^-^/GSH/GPX4 axis was involved in the process.

In summary, the above evidence of *in vitro* and *in vivo* experiments indicated that BBR inhibits the system Xc^-^/GSH/GPX4 axis activation, induces ferroptosis, and prevents NPC cell metastasis. This may be a novel anti-metastatic mechanism of BBR in patients with NPC.

## Figures and Tables

**Table 1:** The primers sequences for qRT-PCR used in this study

Primer	Sequence (5' to 3')	Product size
GPX4 F	CCGCTGTGGAAGTGGATGAAGATC	24
GPX4 R	CTTGTCGATGAGGAACTGTGGAGAG	25
SLC7A11 F	ACGGTGGTGTGTTTGCTGTCTC	22
SLC7A11 R	GCTGGTAGAGGAGTGTGCTTGC	22
SLC3A2 F	TGGGTTCCAGGTTCGGGACATAG	23
SLC3A2 R	TCTGCTGAAGGTCGGAGGAGTTAG	24
GAPDH F	TGACATCAAGAAGGTGGTGAAGCAG	25
GAPDH R	GTGTCGCTGTTGAAGTCAGAGGAG	24
